# How sticky are our proteins? Quantifying hydrophobicity of the human proteome

**DOI:** 10.1093/bioadv/vbac002

**Published:** 2022-01-25

**Authors:** Juami Hermine Mariama van Gils, Dea Gogishvili, Jan van Eck, Robbin Bouwmeester, Erik van Dijk, Sanne Abeln

**Affiliations:** Computer Science Department, Center for Integrative Bioinformatics (IBIVU), Vrije Universiteit Amsterdam, 1081 HV Noord-Holland, The Netherlands

## Abstract

**Summary:**

Proteins tend to bury hydrophobic residues inside their core during the folding process to provide stability to the protein structure and to prevent aggregation. Nevertheless, proteins do expose some ‘sticky’ hydrophobic residues to the solvent. These residues can play an important functional role, e.g. in protein–protein and membrane interactions. Here, we first investigate how hydrophobic protein surfaces are by providing three measures for surface hydrophobicity: the total hydrophobic surface area, the relative hydrophobic surface area and—using our MolPatch method—the largest hydrophobic patch. Secondly, we analyze how difficult it is to predict these measures from sequence: by adapting solvent accessibility predictions from NetSurfP2.0, we obtain well-performing prediction methods for the THSA and RHSA, while predicting LHP is more challenging. Finally, we analyze implications of exposed hydrophobic surfaces: we show that hydrophobic proteins typically have low expression, suggesting cells avoid an overabundance of sticky proteins.

**Availability and implementation:**

The data underlying this article are available in GitHub at https://github.com/ibivu/hydrophobic_patches.

**Supplementary information:**

Supplementary data are available at *Bioinformatics Advances* online.

## 1 Introduction

**Fig. 1.**
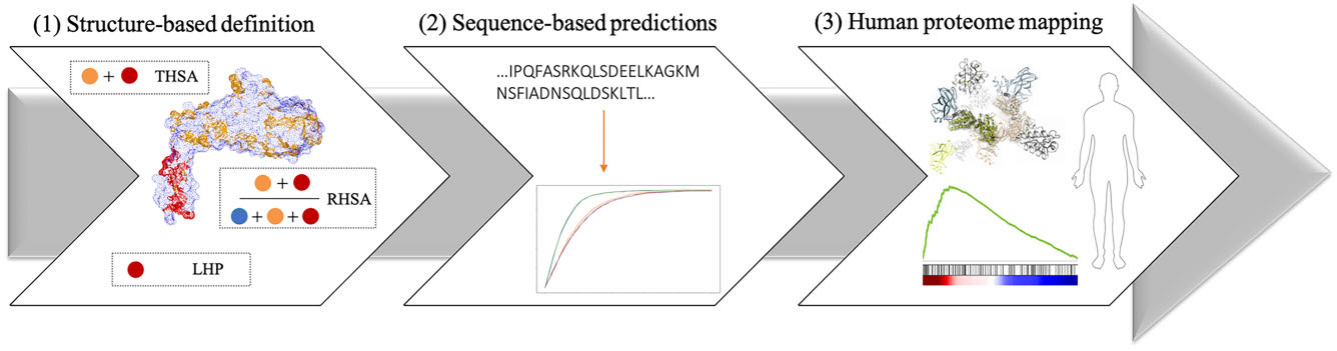
Outline of the study. (**1**) Structure-based definition represents the three hydrophobic measures: red and yellow colours indicate the surface of hydrophobic residues, the blue colour indicates the surface of hydrophilic residues. The THSA is calculated by summing the area of all hydrophobic residues (red and yellow). The RHSA is calculated by dividing the THSA by the TASA (red, yellow and blue). The LHP is the largest area of adjacent hydrophobic residues (only red). (**2**) We train and benchmark sequence-based prediction methods of the three hydrophobic measures. (**3**) THSA, RHSA and LHP values for the human proteome were predicted by the best-performing methods and used to estimate the abundance of hydrophobic proteins in various diseases and tissues

Hydrophobic residues tend to be buried inside the core of a protein to avoid contact with their hydrophilic surroundings (the hydrophobic effect; Dill, 1985, 1990). Hydrophobic residues that do occur on the protein surface often play a functional role, e.g. for protein–protein interactions and membrane binding (Chothia and Janin, 1975; Gowder *et al.*, 2014; Young *et al.*, 1994). Additionally, exposed hydrophobic residues can play a role in the progression of diseases. For example, it has become apparent that hydrophobicity may play a major role in the formation and stabilization of amyloid fibrils (Iadanza *et al.*, 2018; Tuttle *et al.*, 2016; van Gils *et al.*, 2020), which are linked to aggregation diseases such as Alzheimer and Parkinson (Chiti and Dobson, 2006; Dobson, 2001; Koo *et al.*, 1999; Ross and Poirier, 2004). In fact, burying the hydrophobic residues inside the folded protein is also thought to prevent aggregation (Abeln and Frenkel, 2008, 2011; Dobson, 2003). Therefore, hydrophobic patches and residue contributions have been previously used for identifying aggregation-prone regions (Sankar *et al.*, 2018). Abundant exposed hydrophobic residues can also affect experimental outcomes: exposed hydrophobic residues may cause gel formation and prevent crystallization for protein structure determination (Wright and Dyson, 1999); in liquid chromatography surface hydrophobicity is used to separate proteins for further experiments (Moruz and Käll, 2017). All these examples suggests that the more hydrophobic a protein surface, the more ‘sticky’ this protein is to its surrounding (see also panel 1 in Fig. 1). 

The hydrophobic surface area can be defined in different ways. Here, we use three different structure-based measures to describe surface hydrophobicity (see panel 1 in Fig. 1):

1. The total hydrophobic surface area (THSA) is the sum of the exposed surface area of all the hydrophobic residues.
2. The relative hydrophobic surface area (RHSA) is the fraction of the protein surface that is hydrophobic, i.e. the THSA divided by the total accessible surface area (TASA).
3. The largest hydrophobic patch (LHP) is the largest connected hydrophobic area on the protein surface (and is therefore always smaller than or equal to the THSA). It has been shown that LHP size affects protein solubility (Bahadur *et al.*, 2003; Huang and Chandler, 2000; Lijnzaad and Argos, 1997) and function (Dobson, 2004; Larsen *et al.*, 1998).

Note that THSA, RHSA and LHP may not always correlate. For example, a large THSA value can be due to the size of the protein, and a protein with many scattered hydrophobic residues on its surface may have a small LHP but a large THSA and RHSA.

Experimentally, the exposed hydrophobic surface area can be estimated using differential scanning calorimetry, for which the heat capacity temperature relation for the folded protein is directly related to the THSA (Dijk *et al.*, 2016; Gomez *et al.*, 1995).

In this work, our main goal is to investigate *how* hydrophobic protein surfaces are within the human proteome. We also provide some insight how hydrophobicity is related to cellular expression levels, giving an idea of the overall hydrophobicity in the cellular environment. The question *why* some of the human proteome is hydrophobic is not the main focus of our investigation, but is considered in some cases to interpret results.

We use 3D structural information from the Protein Data Bank (PDB) to determine the THSA, RHSA and LHP from structure. The THSA and RHSA can be derived by summing over the exposed surface area per residue calculated by DSSP (Kabsch and Sander, 1983). To calculate the LHP, we introduce a novel method named MolPatch, which is loosely based on a method developed by Lijnzaad *et al.* (1996) and Lijnzaad and Argos (1997).

Since many protein structures have not yet been determined experimentally, we subsequently use the values we obtain from the PDB structures to train/assess predictors for these three hydrophobicity measures. There is a wide range of methods that can predict the surface accessibility for a single residue (Faraggi *et al.*, 2012; Garg *et al.*, 2005; Joo *et al.*, 2012; Klausen *et al.*, 2019; Petersen *et al.*, 2009). However, to predict whether a *hydrophobic* residue will be exposed to the surface is not a trivial task: the earlier methods tended to predict the majority of hydrophobic residues to be fully buried (see Supplementary Fig. S2), as may be expected since the hydrophobicity of residues is strongly associated with being buried inside the protein (Kyte and Doolittle, 1982). The current generation of residue-based surface accessibility predictors use deep neural networks. For example, NetSurfP2.0 is a deep learning-based multitask predictor, which uses evolutionary profiles to make sequence-based predictions of structural features (Klausen *et al.*, 2019). It uses both convolutional and long short-term memory neural layers in the deep learning architecture, with the ability to predict both secondary structure and solvent accessibility (Klausen *et al.*, 2019). Here, we will show NetSurfP2.0 is able to make accurate enough surface accessibility predictions for hydrophobic residues, which in turn can be used to predict the global hydrophobic surface measures described above. Previously, hydrophobic patches have been used to predict the aggregation propensity of protein regions using 3D structures as input (Sankar *et al.*, 2018). This AggScore method, trained on a small (31) number of adnectin proteins, is focused on amyloid-like proteins. In this work, we are interested in generic hydrophobicity of a proteome.

Finally, we use the best-performing prediction methods to predict the THSA, RHSA and LHP of all proteins in the human proteome, and correlate this to cellular expression levels, providing effectively an indication of proteome hydrophobicity per cell type. Subsequently, we use our predictions to provide a glance into the potential implications of a highly hydrophobic proteome in terms of human disease.

## 2 Methods and materials 

**Fig. 2.**
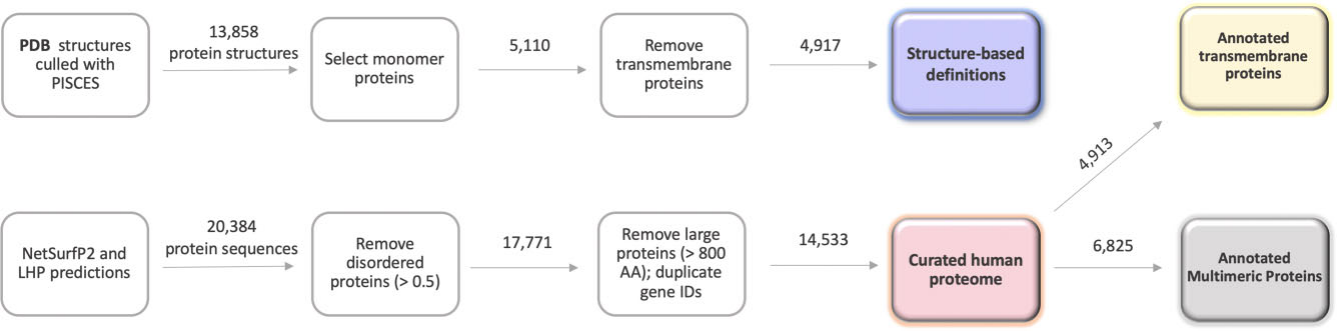
Data curation scheme representing the main steps used to generate datasets for this study. The boxes show the filtering steps and the arrows indicate the number of entries (structures or sequences) passed through. The structure-based definitions dataset used the protein 3D structure information and the human proteome dataset was constructed of protein sequences. The distribution of the measures for surface hydrophobicity within the datasets is represented by the Figure 5 and are colour-coded

Figure 1 indicates how our approach is split into three stages. First, we created a database of filtered PDB structures (Fig. 2) using PISCES (Wang and Dunbrack, 2003). We used this culled set to define measures for surface hydrophobicity: THSA, RHSA and LHP. For the latter, we used a newly developed tool named MolPatch. Second, using the same dataset, we investigated how well we can predict these measures from sequence using the output generated by NetSurfP2.0. Finally, we determined the biological impact of the THSA, RHSA and LHP. To this end, we created a dataset of human proteins from UniProt (Consortium, 2019). We used the best prediction models to predict the THSA, RHSA and LHP for each of these proteins. The structure-based data include protein structures from all organisms, while the sequence-based data only includes human proteins. Subsequently, we correlated gene expression to the hydrophobicity in the human proteome for different cell types.

### 2.1 Calculating the measures for hydrophobicity

To calculate the THSA, we sum over the surface areas of all hydrophobic residues in the protein. For proteins with an available 3D structure in the PDB, this quantity can be determined by calculating the surface area of each residue using DSSP (we used the DSSP module in Biopython version 1.76; Cock *et al.*, 2009). To calculate RHSA, THSA was divided by the total surface area of all residues in the protein. Residues, *r*, were considered hydrophobic in this work, if: r∈{A,C,F,I,L,M,V,W,Y}.

### 2.2 MolPatch

In order to calculate the surface area of the LHP given a protein structure, we need to find the largest connected hydrophobic surface area on the protein’s surface. For this purpose, we developed the tool MolPatch (also see Supplementary Fig. S1). Given the PDB structure of a protein, MolPatch creates a point cloud on the solvent-excluded protein surface (SES) using MSMS (Sanner *et al.*, 1996). In this work, the SES was constructed using a probe of 1.5 Å and a density of 1.5 points per Å^2^. Each point on the point surface was labelled hydrophobic or hydrophilic based on the hydrophobicity classification of the closest *residue*. Initial edges between points were then created if the points existed within a range of 1.25 Å of each other. This search was performed with the KDTree algorithm to speed up the process (Bentley, 1975). Finally, only the edges between node pairs labelled as hydrophobic were retained. This created a network of isolated hydrophobic patches. The individual network components were then extracted for accessible surface area estimation. MolPatch is available on GitHub, this version can also carry out hydrophobic patch identification using atom-based definitions of hydrophobicity for each SES point rather than residue-based definitions. In this work, we only use the residue-based method.

### 2.3 Sequence-based predictions

#### 2.3.1 Data curation

To predict the THSA, RHSA and LHP, a dataset of PDB structures was generated using PISCES. PISCES is a public server for culling sets of protein sequences from the PDB by sequence identity and structural quality criteria (Wang and Dunbrack, 2003). This is important, because using structures with a high sequence identity can introduce bias in the dataset, and factors such as the resolution can affect the accuracy of the results. The chosen parameters were as follows: sequence percentage identity lower or equal to 25%, resolution lower or equal to 3.0 Å, *R*-factor lower or equal to 0.3, sequence length within the range of 40–10 000 amino acids and non-X-ray entries and C*α*-only entries were excluded as we aimed at constructing a homogeneous training dataset for interpretable results. The culled dataset consisted of 13 858 unique protein structures with a selection of 14 604 chains. Two obsolete PDB chains were removed.

In order to avoid bias from hydrophobic patches that may never be exposed in a cellular environment, we filtered multimeric and transmembrane proteins. Multimeric protein structures were filtered out, which resulted in a dataset of 5110 unique monomeric protein structures. Multimers often interact through hydrophobic patches, and if strongly bound such patches will not be exposed in the native environment. Finally, transmembrane proteins have a relatively large hydrophobic surface area. Transmembrane Hidden Markov Model (TMHMM) (Möller *et al.*, 2001; Sonnhammer *et al.*, 1998) was used to filter transmembrane proteins from the dataset (>18 amino acids in transmembrane helices).

#### 2.3.2 Machine learning models

The final dataset for training and testing of the models contained 4917 monomers. For the THSA and RHSA, the values calculated by Define Secondary Structure of Proteins (DSSP) (as described above) were used as training output labels. MolPatch was used to create training output labels for evaluating the LHP predictions. For all models, a training–test split of 80% and 20% was used. Within the training set, a 3-fold cross-validation scheme was used to train the models. Predictions for the THSA, RHSA and LHP were acquired with the following models:

1. The three-feature model (**TFM**) uses the sequence length, number of hydrophobic amino acids and number of hydrophilic amino acids as input. This model is trained using a cubist regression in the CARET module (Kuhn, 2008).
2. The global feature model (**GFM**) uses 31 global features (the total count of each of the 20 possible amino acids in the sequence, sequence length, entropy, hydrophobic amino acid count, polar amino acid count, molecular weight, aromaticity, instability index, gravy score, buried, isoelectric point and molar extinction coefficient) as input. This model is trained using an XGBoost regressor (Chen and Guestrin, 2016).
3. (THSA and RSHA only) NetSurfP2.0 (Klausen *et al.*, 2019) was used to predict the accessible surface area of all the amino acids in a protein. Subsequently, the THSA was calculated by summing over the predicted surface areas of the hydrophobic residues in the protein sequence. The RHSA was calculated by dividing the predicted THSA by the sum of the surface areas of all the residues in the protein as predicted by NetSurfP2.0. Note that we did not perform any model training for this approach; NetSurfP2.0 was used as publicly available with default settings.
4. (LHP only) The LHP cannot be calculated from NetSurfP2.0 predictions directly, as the connections between the residues on the surface are not known from these sequence-based predictions. A random forest model was trained using the RHSA and THSA predicted by NetSurfP2.0 (as described above) as input features. This model was called the NetSurfP-based model (**NBM**).

The data were randomly split into a training and test set of 80% and 20%, respectively. To assess the models, a cross-validation scheme was used. First two-thirds of the training data were used for a 5-fold cross-validation scheme, in which the best-performing parameters for each of the models were optimized using a grid search method. Subsequently the remaining one-third of data, i.e. the validation set, was used to choose the best-performing model. Note that the performance of the models and model parameters was optimized on the *R*^2^. Finally, the 20% of test data was used to estimate the performance of the best-performing model and the NetSurfP2.0 based calculations (code available on GitHub).

#### 2.3.3 Estimation of prediction errors

In order to evaluate the predictions, the structure-based definitions and sequence-based predictions can be compared, by calculating the correlation coefficient *R*^2^. Nevertheless, for difficult regression tasks, this value will put a lot of weight on the outliers, and will not produce results that are easy to interpret. In addition to the *R*^2^ measure, we also evaluated the performance of the prediction model by examining the relative error threshold curve given a certain threshold, partially inspired by the GDT_TS score (Zemla *et al.*, 2001). A major benefit of this method is that it is robust against extreme outliers. For each prediction, the relative THSA error ($\delta_{\mathrm{THS}A_{i}}$), RHSA error ($\delta_{\mathrm{RHS}A_{i}}$) and LHP error ($\delta_{LHP_{i}}$) for each protein *i* are defined by the following formulas:

(1) $$\delta_{\mathrm{THS}A_{i}}=\frac{\left|\mathrm{THS}A_{\mathrm{pre}d_{i}}-\mathrm{THS}A_{\mathrm{DSS}P_{i}}\right|}{\mathrm{THS}A_{\mathrm{DSS}P_{i}}},$$

(2) $$\delta_{\mathrm{RHS}A_{i}}=\frac{\left|\mathrm{RHS}A_{\mathrm{pre}d_{i}}-\mathrm{RHS}A_{\mathrm{DSS}P_{i}}\right|}{\mathrm{RHS}A_{\mathrm{DSS}P_{i}}},$$

(3) $$\delta_{LHP_{i}}=\frac{\left|LHP_{\mathrm{pre}d_{i}}-LHP_{\mathrm{MolPatc}h_{i}}\right|}{LHP_{\mathrm{MolPatc}h_{i}}},$$

where $\mathrm{THS}A_{\mathrm{pre}d_{i}},\;\mathrm{RHS}A_{\mathrm{pre}d_{i}}$ and $LHP_{\mathrm{pre}d_{i}}$ are the predicted THSA, RHSA and LHP of a protein. $\mathrm{THS}A_{\mathrm{DSS}P_{i}}$ and $\mathrm{RHS}A_{\mathrm{DSS}P_{i}}$ are the THSA and RHSA of a protein estimated using DSSP. $LHP_{\mathrm{MolPatc}h_{i}}$ is the predicted LHP of a protein, determined by MolPatch. The performance of the methods over the whole set of structures is evaluated by plotting the percentage correctly predicted instances (protein chains) versus a varying error threshold *t*. The threshold curve, *F*(*t*), shows the percentage of correctly predicted THSA and RHSA of proteins for a given relative error threshold, *t*

(4) $$F\left(t\right)=\frac{\left|\left\{i|i\in \mathrm{chains}\wedge \delta <t\right\}\right|}{\left|\left\{i|i\in \mathrm{chains}\right\}\right|}\cdot 100.$$

The relative error for all chains in the chain dataset is calculated to determine the fraction of correctly predicted chains for the threshold, see also Supplementary Figure S9. The *δ* is here interchangeably used for $\delta_{\mathrm{THS}A_{i}},\;\delta_{\mathrm{RHS}A_{i}}$ or $\delta_{LHP_{i}}$. Unlike in an ROC-curve, the amount of correctly predicted chains does not necessarily have to be 100% when the threshold *t *=* *1.0, since the size of the relative error can be >100%.

### 2.4 Human proteome mapping

#### 2.4.1 Data curation

All reviewed protein sequences for the human genome were extracted from UniProt (Consortium, 2019; accessed 1 October 2020). In total 20 384 sequences were analyzed with NetSurfP2.0 for predicting solvent accessibility and structural disorder among other characteristics. THSA and RHSA values were calculated from NetSurfP2.0 predictions as described above. The LHP for each protein has been predicted using the **NBM**. The following data curation steps have been administered to remove unreliable predictions: (i) highly disordered proteins have been discarded: when more than a half of the residues have been classified as disordered; (ii) large proteins (>800 AA residues) have been discarded in order to match the protein sizes in the structure-based definitions dataset as the majority (99.2%) of proteins are in this range for the training data. (iii) duplicate gene IDs were filtered out and the ones with the highest THSA value were retained. This quality filter resulted in a curated dataset of 14 533 proteins. Separate datasets were created with 4913 proteins annotated as transmembrane and 6825—as multimeric by UniProt (Fig. 2).

Additionally, the final curated dataset described above was used to analyze the link between the expression levels and measures for surface hydrophobicity. RNA consensus tissue gene data were downloaded from Human Protein Atlas (Pontén *et al.*, 2008; Uhlén *et al.*, 2015; accessed on https://www.proteinatlas.org/about/download 24 December 2020). In order to obtain a single expression value for each gene, the highest expression value was selected among all the tissues each gene is expressed in. Subsequently, the genes were divided in deciles based on these values.

#### 2.4.2 Gene Set Enrichment Analysis

To identify tissue types enriched in proteins with large hydrophobic surface area, Gene Set Enrichment Analysis (GSEA) was used. THSA, RHSA and LHP values were centred (such that 0 fell between two parts of a bimodal distribution or between the main bulk and the tail of the distribution, see Supporting Information and Fig. S10) and scaled (Supplementary Fig. S1) prior to the preranked GSEA analysis (Mootha *et al.*, 2003; Subramanian *et al.*, 2005). Tissue-enriched gene sets were downloaded from the Human Protein Atlas (accessed 10 November 2020). 375 disease-associated gene sets were extracted from the GSEA website (accessed on https://www.gsea-msigdb.org/gsea/msigdb/search.jsp 5 November 2020). GSEA was used with the following parameters: number of permutations = 1000; collapse; chip platform: human UniProt IDs MSigDB.v7.2.chip; enrichment statistic: weighted; max size = 1000, min size = 15.

#### 2.4.3 Tissue-specific average surface hydrophobicity

Tissue-specific average surface hydrophobicity (TASH) was calculated across all the genes with the following formula with and without transmembrane proteins

(5) $${\text{TASH}}_{t}=\frac{\sum_{g}{\text{NX}}_{g,t}\cdot h_{g}}{\sum_{g}{\text{NX}}_{g,t}}$$

where ${\text{TASH}}_{t}$ is the TASH for tissue *t*, ${\text{NX}}_{g,t}$ is the normalized expression of gene *g* in tissue *t* and *h* is the predicted hydrophobicity of gene *g* for one of the three measures (THSA, RHSA or LHP). The results are shown in Supplementary Figure S7.

## 3 Results

### 3.1 Structure-based definitions—MolPatch

**Fig. 3.**
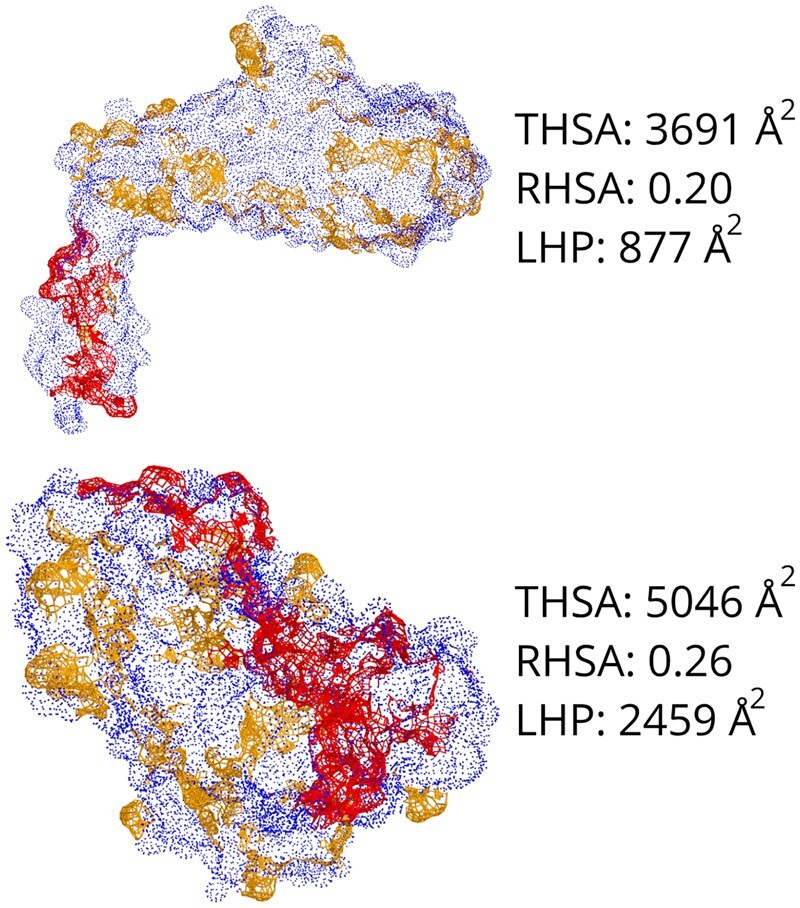
THSA, RHSA and LHP, as identified by MolPatch for two different protein structures. Top: SabA, PDB = 4O5J. Bottom: Leishmanolysin, PDB = 1LML. The surface of hydrophobic residues is displayed in yellow and red. Those in the LHP are displayed in red. The surfaces of the hydrophilic residues are displayed in blue. Note that Leishmanolysin is much larger (465 residues) and has a much larger THSA (5046 Å^2^) compared to SabA (370 residues, 3691 Å^2^), while the RHSA is quite similar between the two proteins, 26% versus 20%. The difference in the LHP is even larger, with 2459 Å^2^ versus 877 Å^2^, respectively; a nearly 3-fold difference

To quantify the exposed hydrophobic areas on the protein surface, we defined three different structure-based measures for surface hydrophobicity, the THSA, RHSA and LHP. Using DSSP (Kabsch and Sander, 1983), we can calculate the THSA and RHSA directly from the surface area per residue (Fig. 3), see methods for further details.

To define the LHP on a protein surface, we developed a novel tool named MolPatch. This tool takes the 3D coordinates in PDB format and identifies networks of adjacent hydrophobic residues to find hydrophobic patches on the protein surface. Hydrophobic patches of 4250 structures of soluble proteins were analyzed using MolPatch (see Section 2). Figure 3 highlights the importance of having three measures by observing the LHP of two proteins with very different surface areas. Although the difference in RHSA between the two proteins is only 6%, the THSA and LHP of Leishmanolysin are approximately 1.5 and 3 times larger than the LHP of SabA. Generally, we see that there is no trivial correlation between THSA, RHSA and LHP (Supplementary Fig. S3).

To determine whether our structure-based largest patch definition is reasonable in biological terms, we overlapped the residues in the 20 LHPs of each protein in our database with those in the PiSITE protein interaction database (also see Supporting Methods). We would expect that large hydrophobic patches, functionally may serve as a protein–protein interaction interfaces. Indeed, we found that overall, the three largest patches in a protein were significantly enriched in protein interaction sites (Supplementary Fig. S4).

### 3.2 Sequence-based predictions—THSA and RHSA can be predicted with reasonable accuracy

Since there are many more protein sequences available than structures, it is highly valuable to be able to predict the THSA, RHSA and LHP from sequence, which will allow us to characterize much broader set of proteins. Thus, we aimed to determine how well we can currently predict the three measures, and identify which sequence features contribute most to the accuracy of these predictions. We used our structure-based definition set to develop sequence-based predictors in a double cross-validation scheme (see Section 2).

**Fig. 4.**
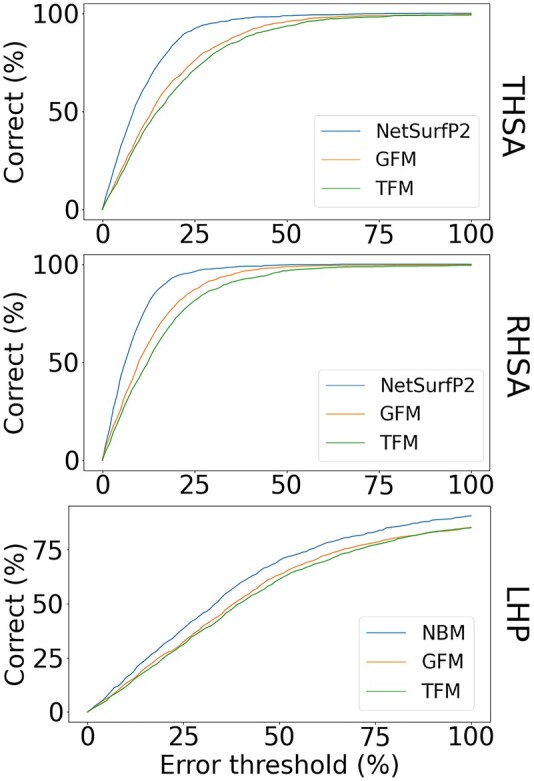
Accuracy of the predictions of the total, relative and largest patch hydrophobic surface area for NetSurfP2.0-based models, the NBM, TFM and GFM. The fraction of correctly predicted proteins within a certain error margin for each of the methods is shown as calculated over the test set

To predict the THSA and RHSA, we used NetSurfP2.0, a neural-network-based method that takes evolutionary conservation profiles as input, and is currently one of the best (non-ensemble) predictors for surface accessibility and secondary structure (Fereshteh *et al.*, 2020; Klausen *et al.*, 2019; Xu *et al.*, 2020). NetSurfP2.0 provides surface area predictions per residue. To obtain the THSA, we summed over the predicted accessible surface areas of all hydrophobic residues. To obtain the RHSA, we summed over the predicted accessible surface area of all residues and divided the THSA by this value. Previous results (see Supporting Information and Fig. S2) indicate that the sequence length and hydrophobicity are strong predictors for the THSA and RHSA, and even outperformed a previous version of NetSurfP2.0 (Supplementary Fig. S5). Therefore, we trained two additional models, one that incorporates the sequence length, the number of hydrophobic residues and the number of hydrophilic residues (three-feature model, TFM), and one that includes a larger number of features derived from the sequence (GFM see Section 2). Figure 4 and Table 1 show that the NetSurfP2.0-based predictions are clearly superior.

**Table 1.** *R* ^2^ of each of the prediction models for the THSA, RHSA and LHP for the four different prediction models as calculated over the test set

	THSA	RHSA	LHP	Features
NetSurfP2.0	0.92	0.77	—	Evolutionary profiles
NBM	—	—	0.43	THSA and RHSA predictions by NetSurfP2.0
TFM	0.71	0.13	0.00	Sequence length, number of hydrophobic residues, number of hydrophilic residues
GFM	0.75	0.49	0.12	31 sequence-based features

*Note*: For all three measures, the NBM/NetSurfP2.0 models performed significantly better than the other models [*P* < 0.001, *t*-test for correlations (Fisher, 1921)].

The TFM, which only includes the features sequence length, number of hydrophobic and number of hydrophilic residues, also performs significantly better than random for both the THSA and RHSA, indicating that these features are of major significance for predicting these two properties. The GFM, which includes 31 features, performs only marginally better than the TFM, indicating that sequence length and sequence hydrophobicity are some of the main determinants for the hydrophobic surface area. Since it is difficult to obtain feature importance from neural network models such as NetSurfP2.0, we also analyzed the feature importance measures from the GFM. This analysis showed that the hydrophobicity of the sequence is another major predictor for the THSA and RHSA [Supplementary Fig. S5, gravy score (Kyte and Doolittle, 1982), aromaticity (Lobry and Gautier, 1994), hydr_count].

To predict the LHP from sequence, the LHP determined by MolPatch was used as a gold standard. The training procedure for the TFM and GFM for predicting the LHP was performed in a similar fashion to the training for THSA and RHSA. Since the NetSurfP2.0 predictions cannot readily be used to predict the LHP, a model was trained that uses the THSA and RHSA predicted by NetSurfP2.0 as input features to predict the LHP (NetSurfP-based model, NBM). The results are shown in Figure 4 and Table 1. One can see that the NBM outperforms the other two methods. The sequence hydrophobicity again appears to have a major contribution to the prediction results (Supplementary Fig. S5). Nevertheless, each of these prediction models perform significantly worse than the models for the THSA and RHSA predictions (Table 1), suggesting LHP prediction is less straightforward than THSA or RHSA predictions.

### 3.3 Human proteome mapping

#### 3.3.1 Transmembrane proteins—the most hydrophobic part of the human proteome

**Fig. 5.**
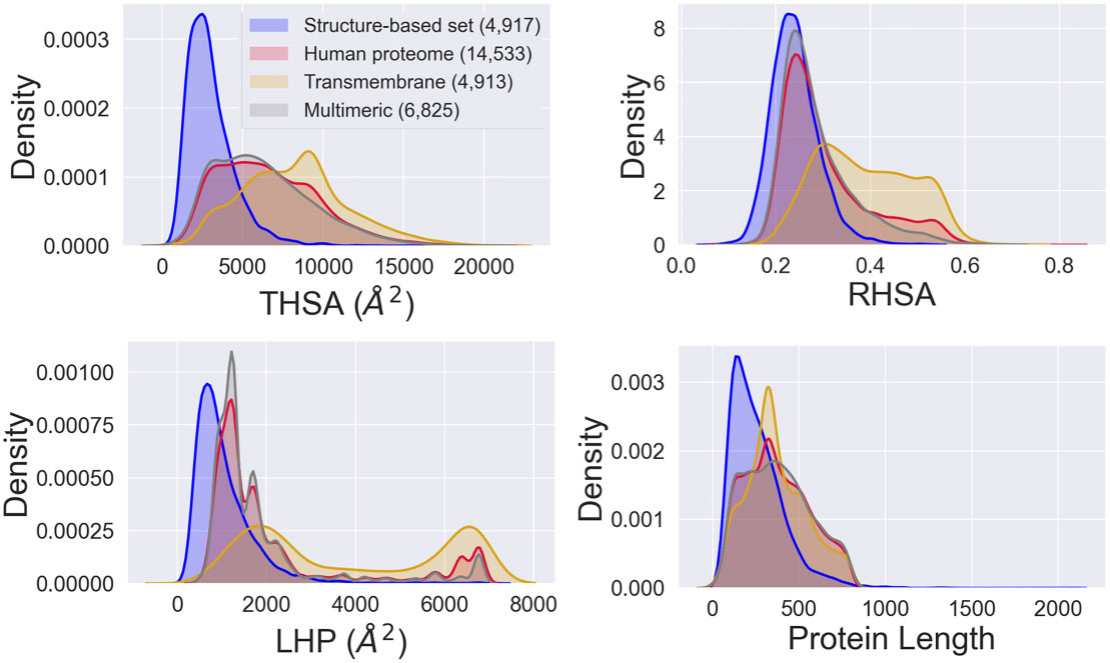
The distribution of the protein length, THSA, RHSA and LHP values from the whole curated human proteome (red, predicted values), annotated transmembrane (yellow, predicted values) and multimeric (grey, predicted values) proteins and the same values in the structure-based dataset (blue, structure-based values) for the comparison. The structure-based dataset generally contains smaller proteins than the human proteome datasets, as may be observed from the length distribution. The numbers in brackets in the legend indicate the sizes of the datasets analyzed. The figure also shows that transmembrane proteins are predicted to have large hydrophobic surface areas, as can be observed in the LHP plot: ∼2000 Å^2^; ∼6500 Å^2^

For 14 533 proteins in the human proteome, we were able to predict THSA, RHSA and LHP values (see Section 2). Figure 5 shows a comparison of the distributions of the definitions of these values on the structural dataset and of the predicted values on the human proteome dataset. One can see from the figure that proteins in the structure-based dataset appear to be smaller compared to those in the curated human proteome. In line with this, we see that the predicted THSA and LHP distributions are strongly shifted towards the right-hand side compared to the structure-based data, most likely due to the larger size of proteins in the human proteome.

Moreover, the structure-based set (blue) does not show a peak of very large hydrophobic patches (LHP, ∼6500 Å^2^) as observed for the human proteome dataset (red). Importantly, structure-based data analyzed by MolPatch neither contain proteins with more than one chain in the PDB structure nor transmembrane proteins; both groups of proteins maybe expected to have a very large hydrophobic patch. To investigate if this peak for the human proteome may be due to transmembrane or multimeric proteins, we selected those proteins annotated by UniProt (Consortium, 2019) as ‘transmembrane’ (yellow), or ‘part of the protein complex’ (grey). Note that these sequence-based curated UniProt annotations are not identical to the structure-based exclusion criteria for the training dataset (see Section 2).

Indeed, when comparing transmembrane proteins from the human proteome dataset to the predictions for the entire human proteome, the composition of peak of the large hydrophobic patches, as well as the shoulder in the RHSA distribution can be explained predominantly through the transmembrane annotated proteins (Fig. 5). Note that the two distinct peaks in the predicted LHP for the transmembrane proteins can be explained by the size of the transmembrane domain: some proteins contain a large transmembrane domain (LHP ∼ 6500 Å^2^), while other proteins are anchored to the membrane with a single transmembrane helix (LHP ∼ 2000 Å^2^). THSA and RHSA do not show two distinct peaks for the LHP, instead we see a shoulder at higher values—corresponding to large transmembrane domains.

Multimeric proteins mostly follow the distribution of the whole human proteome and do not appear to be much more hydrophobic in general. The results in Figure 5 also suggest that our ML model (NBM) successfully predicted transmembrane proteins to have large hydrophobic patches, despite the lack of transmembrane proteins in the training dataset.

#### 3.3.2 Cells avoid the over expression of proteins with a large hydrophobic surface area

**Fig. 6.**
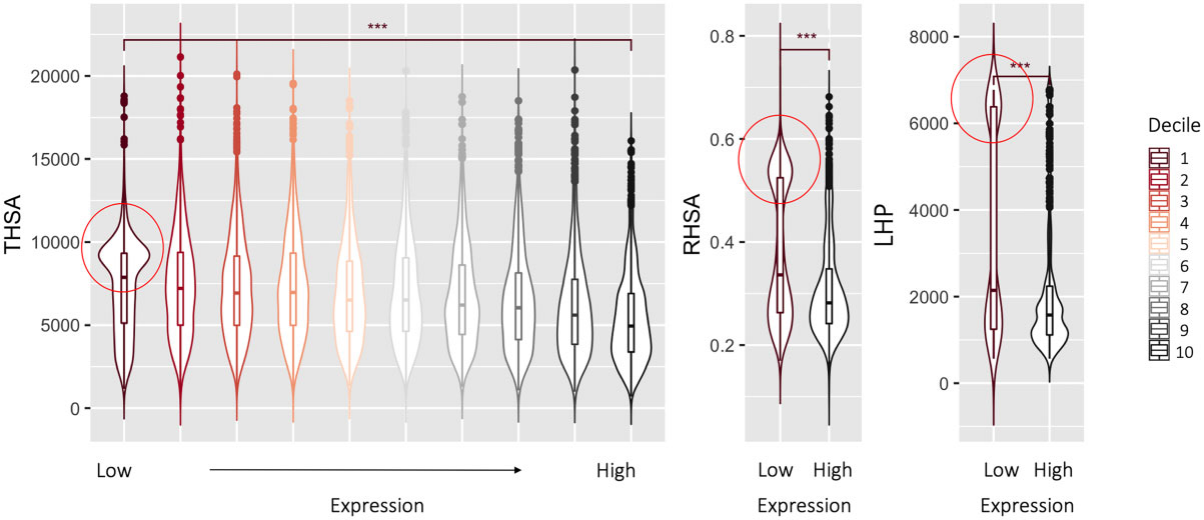
Relationship between normalized expression (NX) and THSA, RHSA and LHP values. For each gene, the highest NX value was selected across all tissues. The genes were grouped in 10 bins based on their expression levels. Each violin represents one decile of genes grouped by their normalized expression values (*x* axis). Boxplots inside the violins show the median and quartiles for the surface hydrophobicity measures (*y* axis). As presented in the legend, dark red indicates the decile with the lowest NX values and black indicates the decile with the highest NX values. The deciles with the lowest NX values show significantly higher THSA, RHSA and LHP values (indicated by red circles), compared to the deciles with the highest NX values. Significance was calculated using Wilcoxon signed-rank test and shown between the lowest and the highest expression deciles. The three asterisks indicate *P*-values <2.22e−16. Note that the intermediate groups in the case of RHSA and LHP are excluded from the plot, as they show the similar trend to the THSA

Since hydrophobic characteristics are associated with the aggregation tendencies, we wanted to investigate whether proteins with large hydrophobic surface areas have different expression levels. We used the RNA consensus tissue gene data from the Human Proteome Atlas to explore a link with expression levels. For this, we relate normalized expression (NX) data to measures for surface hydrophobicity. To obtain a single expression value for each gene we took the highest expression value among all the tissues each gene is expressed in, and subsequently divided the genes into deciles based on these values. Figure 6 shows the lowest and highest deciles for each of the hydrophobic measures. When comparing the lowest and highest deciles, it is clear that only in the lowly expressed proteins, a group of proteins with very hydrophobic surfaces is present (red circles in Fig. 6).

Since it is not trivial to define a measure of expression across different tissue types, we also explored the a median NX value across all the tissues that a particular gene appears in. The resulting deciles show a similar trend in terms of hydrophobicity of the surface (see Supplementary Fig. S6) compared to the data based on the highest NX value. Interestingly, proteins that do not follow the general trend, i.e. those that are highly expressed while having a large THSA, RHSA and LHP value, are typically protein subunits assembling large multimeric complexes. In such complexes, the proteins are likely to be stably bound, and are hence able to shield the hydrophobic surfaces from the solvent.

#### 3.3.3 The brain- and kidney-specific proteomes are enriched with hydrophobic proteins

To investigate if genes that are enriched in specific tissues are associated with the hydrophobic properties of the proteins, we carried out GSEA. We downloaded five tissue-enriched gene sets from the Human Protein Atlas (HPA; Pontén *et al.*, 2008; Uhlén *et al.*, 2015). Table 2 shows that the brain tissue-enriched gene set has a high enrichment in predicted THSA and LHP values. Kidney-enriched genes show the highest enrichment in THSA, RHSA and LHP of the ranked gene lists (*P*-values <0.001). A possible explanation for this is the major role of kidney tissue in maintaining homeostasis through various membrane-bound receptors and transporters (Lote, 1994). Indeed, 79% of the kidney-enriched proteome is annotated as transmembrane by UniProt (Consortium, 2019). Interestingly, liver tissue revealed no enrichment. The skin and blood tissue-enriched gene sets exhibited significant enrichment in the RHSA ranked list. Furthermore, both tissue groups were significantly depleted in the THSA ranked list, indicating that they may contain the smaller proteins in the human proteome.

**Table 2.** Preranked GSEA enrichment statistics in different tissues

Gene set	ES (THSA	ES (RHSA)	ES (LHP)	TM (%)	Multimeric (%)
Brain (488)	0.33^**^	0.14	0.64^**^	47.0	47.0
Kidney (53)	0.62^**^	0.53^**^	0.78^**^	79.2	35.8
Skin (113)	−0.46^*^	0.30^**^	0.44	7.9	15.9
Liver (242)	−0.22	−0.16	0.45	26.0	59.9
Blood (57)	−0.41^*^	0.40^**^	0.68^*^	47.4	28.0

*Note*: Various tissue-enriched gene sets were obtained from the HPA (Pontén *et al.*, 2008; Uhlén *et al.*, 2015). THSA, RHSA and LHP values were central-scaled prior to the GSEA analysis. The enrichment score (ES) is the maximum deviation from zero showing the degree to which the gene set is over-represented at the top (positive ES score) or bottom (negative ES score) of the entire ranked list of genes. The fraction of transmembrane (TM) and multimeric proteins in the following gene sets is shown in percentages.

* *P* < 0.05.

** *P* < 0.001.

To investigate the overall tissue hydrophobicity, we introduced TASH for *all proteins* based on the expression levels in a specific tissue (Equation 5 and Supplementary Fig. S7). The TASH-THSA value provides an indication of the THSA present in a specific cell type. The tissues with the highest TASH-THSA values occur in the brain, such as the cerebellum, corpus callosum, thalamus, cerebral cortex and basal ganglia (Supplementary Fig. S7).

#### 3.3.4 Increased relative hydrophobicity is associated with (aggregation) diseases

To investigate the association of surface hydrophobicity with human diseases, a GSEA preranked analysis of 375 various disease-associated gene sets was carried out, of which 44 gene sets show a significant (*P*-value <0.05) enrichment [<−0.2 (negative enrichment) and >0.2 (positive enrichment)] in at least two hydrophobic measures (see Supplementary Fig. S8). Among the enriched gene sets, we can observe several KEGG (Kanehisa and Goto, 2000) pathways that are associated with neurological disorders. The RHSA showed a significant (*P*-value <0.05) enrichment in Parkinson’s (ES = 0.43), Alzheimer’s (ES = 0.24) and Huntington’s disease (ES = 0.23) gene sets. The analysis shows a significant (*P*-value <0.001) enrichment of sticky proteins (based on LHP) in the KEGG Parkinson’s disease map (ES = 0.66). In contrast to the GSEA analysis results on tissue-specific proteome, the THSA shows a negative enrichment in these sets, suggesting that the proteins involved in pathological pathways have large hydrophobic surfaces and patches, but are smaller in size (median length 171–180 residues).

## 4 Discussion

In this work, we analyzed the predictability of hydrophobic areas on protein surfaces, which until recently was a difficult problem. We show that THSA and RHSA values can be predicted with high accuracy (>75% within a 20% error margin, Fig. 4). The improved predictions of NetSurfP2.0, compared to the earlier secondary structure prediction methods (Supplementary Fig. S2), make this possible by straightforward calculations of the THSA and RHSA using the predictions of the surface accessibility per residue from NetSurfP2.0. Note that the problem of predicting the THSA, RHSA and LHP for a protein sequence, means we are trying to predict a global feature of the protein, while NetSurfP2.0 (Klausen *et al.*, 2019) makes residue-based predictions; residue-based models can be trained on many more labels that are available from protein structures, and hence can reach a richer model representation.

On the other hand, the LHP cannot be directly obtained from NetSurfP2.0 (Klausen *et al.*, 2019) and needs additional model training. The major difficulty herein is that NetSurfP2.0 cannot predict which hydrophobic residue form a continuous patch in the protein 3D structure. Nevertheless, we believe that recent advances in deep neural nets, contact map prediction and structure prediction (Li *et al.*, 2019; Senior *et al.*, 2020; Xu *et al.*, 2020; Zheng *et al.*, 2019) should make it possible to make these predictions more accurate in the near future, e.g. by using structure or contact predictions to predict the hydrophobic patches, or by training a purpose specific deep neural net. Recently, language models, especially bidirectional encoder representations from transformers (BERT), have been shown to be a promising model achieving novel state-of-the-art performance (Shah and Ou, 2021). BERT adopted the concept of contextualized word embedding to capture the semantics and sequence information, opening a new avenue in biological modelling. Such models trained on LHP data per residue might also lead to a better performance in predicting such a complex characteristic.

When investigating the link between tissue-based expression levels and the measures for surface hydrophobicity, we clearly observe that highly expressed proteins typically do not have a large hydrophobic surface area (THSA, RHSA and LHP as seen in Fig. 6). A similar trend has previously been observed for proteins with a strong tendency to form amyloid fibrils (Tartaglia *et al.*, 2009), suggesting an evolutionary pressure to avoid proteins with high aggregation propensities being present at high concentrations in the cell. Based on our data, if we assume that the high expression values correlate with high protein abundance in the cell, it is conceivable that there is also an evolutionary pressure against proteins with a large hydrophobic surface area to be overly abundant in the cell.

Note that while the THSA and RHSA sequence-based predictions show a reasonable correlation with the structure-based definitions, this does not necessarily mean that the predicted amount of accessible hydrophobic surface area is actually exposed to the cellular environment. For example, a hydrophobic patch may be buried in a stable macromolecular complex, or may be buried inside a membrane. Additionally, a high hydrophobic surface area does not necessarily mean a protein will be insoluble; this will also be very much dependent on the amount of polar and charged residues that may surround the hydrophobic residues or patches (Kramer *et al.*, 2012), as well as disordered regions (Abeln and Frenkel, 2008).

Despite the general tendency to avoid highly expressed proteins with a large hydrophobic surface area, the brain appears to be highly hydrophobic in its overall expression patterns (THSA in cerebellum, cerebral and cortex as shown in Supplementary Fig. S7) and in proteins enriched in the brain (THSA and LHP as shown in Table 2). This high expression of proteins with a large hydrophobic surface area may be rationalized by functional requirements: genes enriched in brain tissue are involved in organizing and maintaining synaptic signalling, requiring various cell adhesion proteins with large hydrophobic surface areas (Sytnyk *et al.*, 2017); the cellular morphology of neurons including the dendrite means that there is a relatively large transmembrane surface area per cell. Additionally, the structural integrity of neuronal axons is facilitated by myelin (Stadelmann *et al.*, 2019), a fatty substance surrounding neurons, and by myelin-associated proteins, which are all very hydrophobic.

Furthermore, brain tissue has been associated with various aggregation diseases (Chiti and Dobson, 2006; Dobson, 2001; Koo *et al.*, 1999; Ross and Poirier, 2004). Based on our data, it may be hypothesized that the brain is specifically vulnerable to such diseases due to its high expression of proteins with a large hydrophobic surface. Hydrophobic patches play a role in the folding and/or misfolding of proteins (Dobson, 2004; Ross and Poirier, 2004), and can possibly provide nucleation sites for the formation of oligomers and amyloid fibrils. This hypothesis would be supported by the relatively high hydrophobic surface area in molecular pathways associated with Parkinson’s, Huntington’s and Alzheimer’s disease (as observed for the RHSA and LHP, see Supplementary Fig. S8).

## 5 Conclusion

In conclusion, in this work, we defined three measures for hydrophobicity: the THSA, RHSA and LHP. To determine the LHP from structure, we developed a novel method named MolPatch. The THSA and RHSA can be accurately predicted from sequence by adapting the output from NetSurfP2.0, while predicting the LHP from sequence remains challenging. We have investigated the potential impact of the three measures by investigating the relation between these measures and protein expression in the human proteome. Cells tend to avoid high expression of proteins with a large amount of hydrophobic surface, probably to reduce the risk of protein aggregation or unspecific binding. However, some tissue types have a relatively hydrophobic environment, like the brain. In these tissues, proteins with a strongly hydrophobic surface are more common. This may perhaps explain why the brain is especially prone to aggregation diseases.

## Supplementary Material

vbac002_Supplementary_Data
