# Supplementary material for: How sticky are our proteins? Quantifying hydrophobicity of the human proteome
Source: Bioinform Adv. 2022 Jan 25;2(1):vbac002. doi: 10.1093/bioadv/vbac002 (PMC9710682; doi:10.1093/bioadv/vbac002)
Supplement: vbac002_Supplementary_Data [file vbac002_supplementary_data.pdf]

Supporting Information

Centering and scaling of the THSA, RHSA and LHP distributions for GSEA

THSA, RHSA, and LHP values were centered and scaled prior GSEA analysis. To get biologically meaningful values, the values for centering were chosen such that 0 fell in between two parts of a bimodal distribution or between the main bulk and the tail of the distribution S10:

$$x_{i_{new}} = \frac{x_{i_{old}} - center}{\sigma} \tag{S1}$$

where $x_{i_{new}}$ is the centered and scaled value for protein i, $x_{i_{old}}$ is the original value (THSA, RHSA or LHP) of protein i and center is the zero position chosen based on the original distributions: 8106 Å for the THSA, 0.35 for the RHSA and 1656 Åfor the LHP, see Figure S10.

Benchmark of older methods

Previous (unpublished) results of a benchmark of the TFM against SANN (Joo et al., 2012), NetsurfP (NOT the 2.0 version) (Petersen et al., 2009), SARPRED (Garg et al., 2005), SPINEX (Faraggi et al., 2012) and a simple length-based reference model for predicting the THSA, as shown in Figure S2.

SANN, NetsurfP, SARPRED and SPINEX were run using their default setting. Each of these methods predicts the surface area per residue. We summed over the predictions of all the hydrophobic residues to obtain the THSA.

For the purpose of model comparison, we also developed a length-based reference model. This simple model provides an HSA estimate based on the length of the protein sequence. The idea of approximating proteins as a sphere to predict the ASA of the whole protein was first introduced in (Janin, 1979). The ratio between hydrophilic and hydrophobic residues on the surface has previously been observed in (Chothia, 1976): for proteins with a high molecular weight the ratio of hydrophobic residues can be well approximated for globular proteins based on the length of the protein sequence alone.

The reference model uses the sequence length of a protein (L) multiplied with a constant (k_1) and to the power of a constant (k_2) to predict the HSA:

$$ASA = k_1 \cdot L^{k_2} \tag{S2}$$

Note that in case of a perfect sphere, we would have:

$$\text{surface area} = 4\pi \left(\frac{3V}{4\pi}\right)^{\frac{2}{3}} \tag{S3}$$

Using the latter equation the total ASA could be approximated by assuming the sequence length (L) scales linearly with the volume (V). However, since proteins are not perfect spheres and only a fraction of the surface is covered by hydrophobic groups, we instead generate the baseline model by fitting the constants k_1 and k_2 to the training set, minimising the sum of squares between the predicted and observed HSA. In this simple model, we effectively assume that the fraction of hydrophobic amino acids on the surface with respect to the length is constant.

Surprisingly, the TFM outperforms all other methods including NetsurfP (Petersen et al., 2009), which incorporates more information (evolutionary profiles) and has a more complicated architecture (neural network).

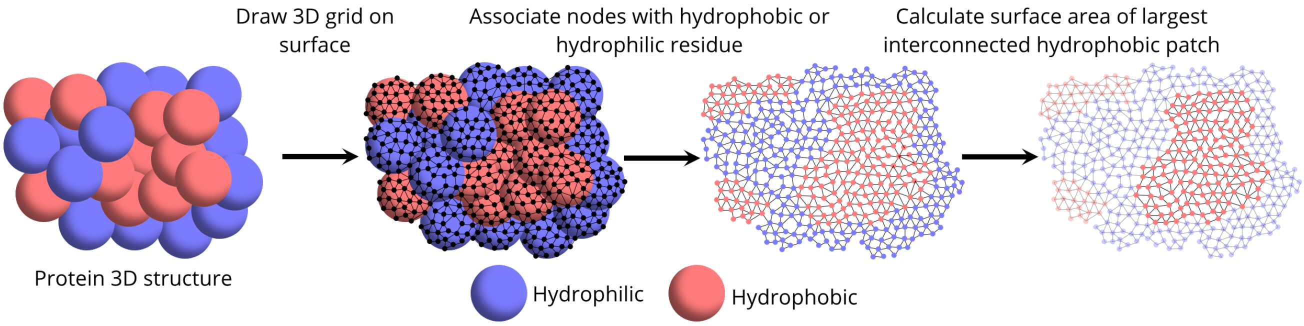

Fig. S1. MolPatch workflow. MolPatch takes a protein 3D structure in PDB format, and places nodes on the surface of this protein. Each node is considered hydrophobic or hydrophilic depending on its nearest residue. Note that MolPatch, as available on github, can also do this at atom level. Nodes are connected if they are within a set distance from each other to create a network. Subsequently, the method identifies hydrophobic patches by searching for groups of interconnected hydrophobic nodes. Finally, it calculates the surface area of the largest hydrophobic patch.

Supplementary figures

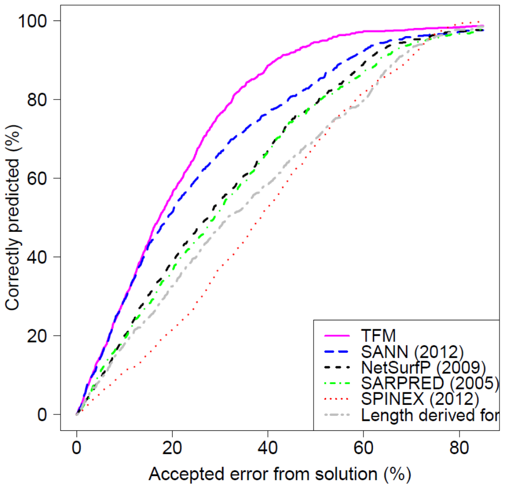

Fig. S2. Benchmark of SANN, NetsurfP, SARPRED, SPINEX and LBM for hydrophobic surface area predictions. The figure shows that the TFM outperforms the other methods, indicating that the length and hydrophobicity of the sequence are very important features for predicting the surface area.

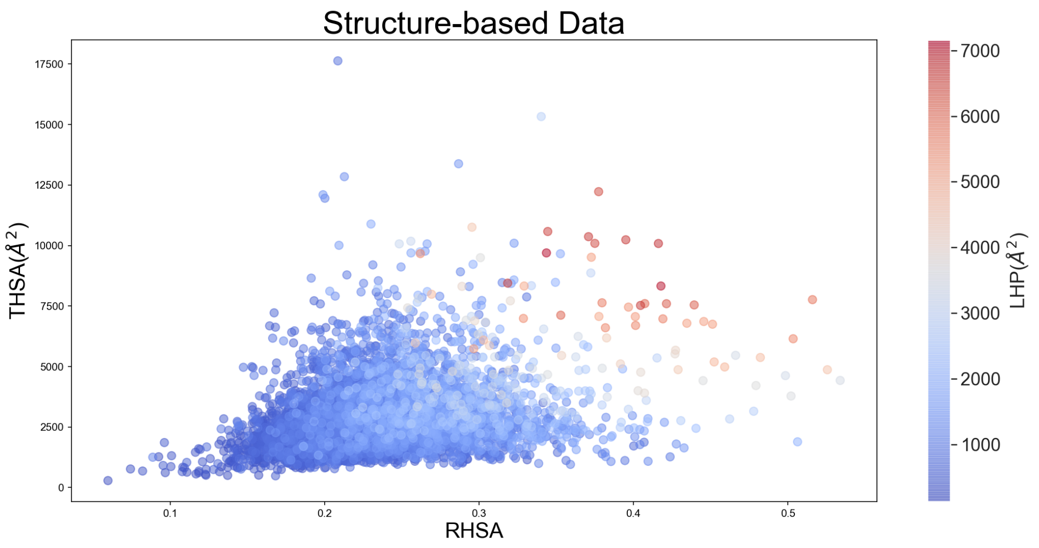

Fig. S3. Scatter plot showing the distribution of proteins in the structure-based dataset based on THSA and RHSA values. LHP values are colour-coded.

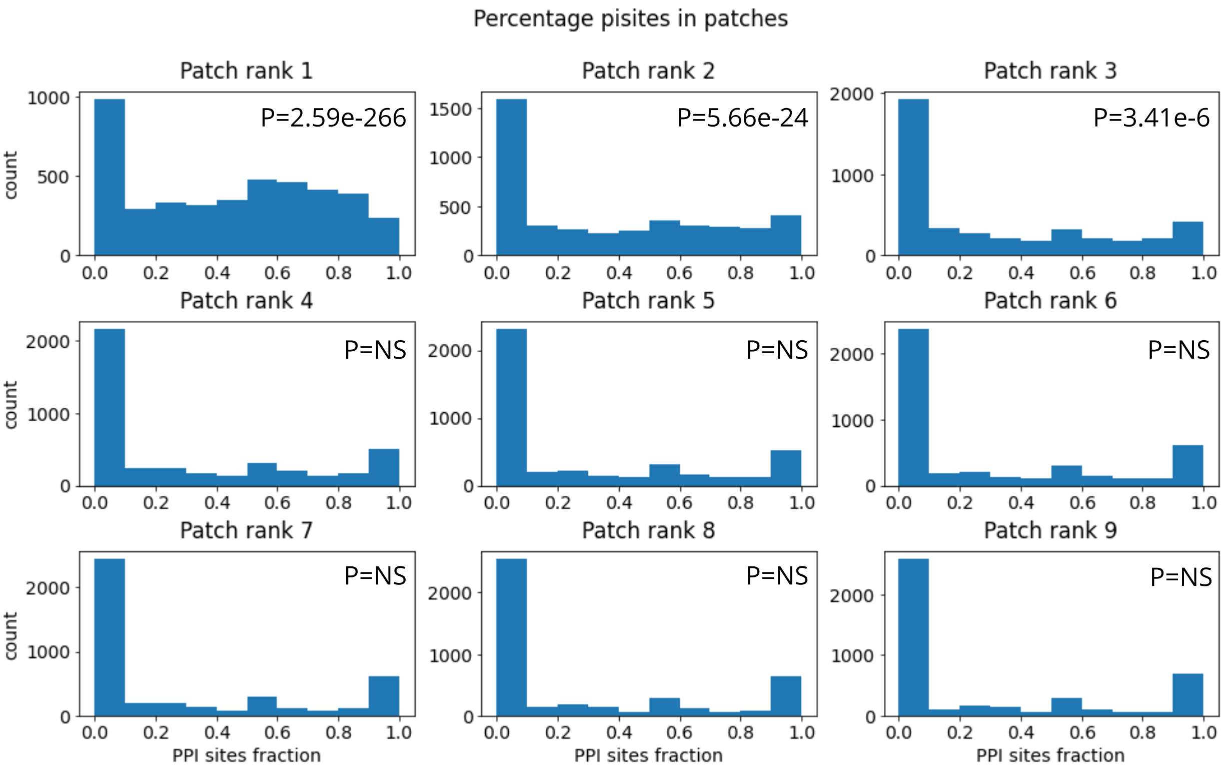

Fig. S4. Distribution of interaction sites over the nine largest hydrophobic patches per protein. For each patch on a protein we calculated which fraction of the hydrophobic patch on that protein overlaps with protein-protein interaction sites for the nine largest hydrophobic patches. The three largest patches in each protein have a significantly larger overlap with the PPIs compared to the general surface residues. The dataset with the information about protein binding sites was obtained via the PiSITE database. Both interaction information from a single PDB complex and interaction information between multiple PDBs are stored (Higurashi et al., 2009). Only PiSITE information of the proteins from the original 14,602 chains dataset was included. The proteins without interaction sites and transmembrane proteins were filtered, which resulted in the dataset of 4,255 entries with information about protein interaction sites. NS = not significant.

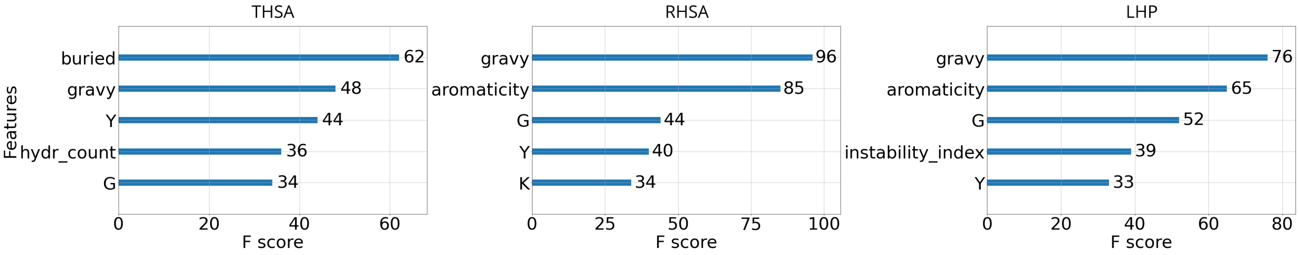

Fig. S5. Feature importance of the GFM for the THSA, RHSA and LHP predictions. The five most important features in the GFM for each of the measures of surface hydrophobicity were extracted using the XGBoost Python package (Chen and Guestrin, 2016). The letters represent amino acids. A higher F score indicates a higher importance. One can see that in all cases buriedness and/or hydrophobicity (hydr_count, gravy, aromaticity (Kyte and Doolittle, 1982a; Lobry and Gautier, 1994)) is important for the predictions. Note that since hydrophobic residues tend to be buried, the feature 'buried' (sum over the average area buried upon folding for each amino acid), which is the most important feature for the THSA predictions, is related to hydrophobicity.

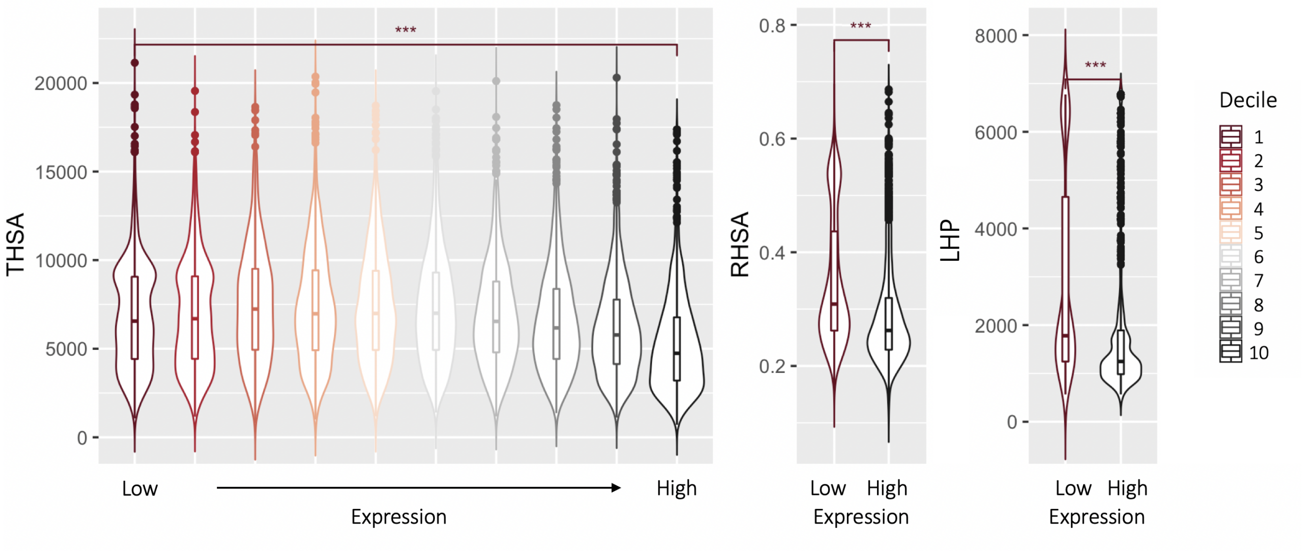

Fig. S6. The figure shows the relationship between normalised expression (NX) and THSA, RHSA and LHP values respectively. The median NX value per gene was calculated across all tissues. The genes were grouped in ten bins based on their expression levels. Each violin represents one decile of genes grouped by their normalised expression values (x-axis). Boxplots inside the violins show the median and quartiles (y-axis) of the surface hydrophobicity measures. As presented in the legend, dark red indicates the decile with the lowest NX values and black indicates the decile with the highest NX values. The deciles with the lowest NX values show significantly higher THSA, RHSA and LHP values compared to groups with the highest NX values. Significance was calculated using Wilcoxon signed-rank test and shown between the lowest and the highest expression deciles. The three asterisks indicate p-values<2.22e-16. Note that the intermediate groups in the case of RHSA and LHP are excluded from the plot, as they show the similar trend to the THSA.

TISSUE	TASH_THSA	TISSUE	TASH_RHSA	TISSUE	TASH_LHP
parathyroid gland	6589.344140	salivary gland	0.303269	granulocytes	2065.153865
cerebellum	6543.820250	granulocytes	0.302792	small intestine	2062.743761
corpus callosum	6406.807438	pancreas	0.298075	salivary gland	2050.500048
retina	6376.943688	epididymis	0.296657	kidney	2035.300969
placenta	6345.451364	dendritic cells	0.295775	retina	2026.744680
thalamus	6324.745924	monocytes	0.295740	lung	2012.001148
cerebral cortex	6320.775118	kidney	0.295681	cerebral cortex	1994.404812
skin	6318.893971	small intestine	0.294872	corpus callosum	1992.950579
pituitary gland	6277.653711	liver	0.294281	placenta	1986.760278
testis	6273.384076	lung	0.293951	duodenum	1986.178344
basal ganglia	6270.733428	heart muscle	0.293544	liver	1973.574641
small intestine	6268.261355	retina	0.291871	epididymis	1973.078645
pons and medulla	6237.357534	B-cells	0.291560	spinal cord	1972.529287
endometrium	6220.467402	duodenum	0.291439	basal ganglia	1966.613790
lung	6199.512757	placenta	0.291329	colon	1965.886036
seminal vesicle	6195.152068	stomach	0.291204	adipose tissue	1957.522483
spinal cord	6177.328519	adipose tissue	0.290725	midbrain	1956.156072
cervix, uterine	6168.331312	colon	0.290702	seminal vesicle	1954.696576
gallbladder	6164.707883	adrenal gland	0.290092	parathyroid gland	1954.094850
hippocampal formation	6164.486632	gallbladder	0.290080	adrenal gland	1951.654898
prostate	6154.472076	total PBMC	0.289878	hippocampal formation	1949.681780
fallopian tube	6152.572096	T-cells	0.289791	pancreas	1949.002380
adipose tissue	6149.651771	ductus deferens	0.289610	monocytes	1948.151491
spleen	6147.649362	seminal vesicle	0.289535	hypothalamus	1947.830139
ductus deferens	6146.424261	spinal cord	0.289143	stomach	1947.377945
thyroid gland	6145.629037	midbrain	0.289017	ductus deferens	1944.970632
appendix	6144.180903	cerebral cortex	0.289007	gallbladder	1943.780472
midbrain	6143.051861	hypothalamus	0.288903	pons and medulla	1938.526900
duodenum	6130.972608	basal ganglia	0.288845	heart muscle	1932.798900
colon	6128.840459	breast	0.288750	dendritic cells	1927.906919
olfactory region	6127.506844	hippocampal formation	0.288708	amygdala	1926.043147
thymus	6114.352108	rectum	0.288555	thyroid gland	1920.606895
kidney	6106.684832	cervix, uterine	0.288112	rectum	1920.109998
stomach	6102.693786	corpus callosum	0.288077	cervix, uterine	1919.336611
urinary bladder	6098.241063	amygdala	0.288054	breast	1910.694675
adrenal gland	6096.653533	thyroid gland	0.287993	spleen	1910.191559
amygdala	6073.329105	NK-cells	0.287912	prostate	1907.293782
smooth muscle	6060.004560	spleen	0.287098	skin	1903.637730
vagina	6058.344196	prostate	0.286653	thalamus	1881.143970
ovary	6056.220191	pons and medulla	0.286649	pituitary gland	1878.883236
tonsil	6052.353966	esophagus	0.286528	esophagus	1875.343994
hypothalamus	6049.555798	lymph node	0.286512	lymph node	1873.364843
rectum	6020.849246	skin	0.286394	tonsil	1862.685347
breast	5994.053978	tongue	0.285365	cerebellum	1861.579370
esophagus	5983.638528	skeletal muscle	0.285309	B-cells	1860.039040
liver	5973.750280	parathyroid gland	0.284993	urinary bladder	1859.762336
epididymis	5943.171691	pituitary gland	0.284332	fallopian tube	1857.669888
salivary gland	5905.473189	tonsil	0.284184	appendix	1853.751783
lymph node	5857.352586	urinary bladder	0.284013	T-cells	1853.746152
heart muscle	5841.167449	fallopian tube	0.283914	olfactory region	1852.028624
granulocytes	5737.364121	appendix	0.283111	endometrium	1847.616184
skeletal muscle	5727.633148	endometrium	0.282286	testis	1838.423460
bone marrow	5716.439895	thalamus	0.282099	NK-cells	1838.142712
tongue	5682.001577	olfactory region	0.281939	smooth muscle	1808.947763
pancreas	5560.585334	testis	0.281936	tongue	1805.763883
NK-cells	5488.137137	smooth muscle	0.281307	vagina	1799.745722
monocytes	5470.279925	bone marrow	0.280638	skeletal muscle	1795.502473
T-cells	5390.961264	ovary	0.279915	ovary	1789.910836
dendritic cells	5360.232129	vagina	0.279811	total PBMC	1789.443529
B-cells	5274.924184	cerebellum	0.279514	bone marrow	1772.438651
total PBMC	4865.713664	thymus	0.275642	thymus	1745.731552

Fig. S7. Tissue-specific average surface hydrophobicity calculated (Equation 5) for different hydrophobic measures. Each column is independently sorted and colour-coded based on TASH values.

Disease Gene Set	ES_THSA	p-val	ES_RHSA	p-val	ES_LHP	p-val
KEGG_PARKINSONS_DISEASE	-0.414753	0.000000	0.434188	0.000000	0.659828	0.000000
CAMPS_COLON_CANCER_COPY_NUMBER_DN	nan	nan	0.415266	0.000000	0.684271	0.000000
HP_MITOCHONDRIAL_MYOPATHY	nan	nan	0.404508	0.000000	0.673684	0.003003
KEGG_AUTOIMMUNE_THYROID_DISEASE	nan	nan	0.347517	0.000000	0.630180	0.037074
HP_NEPHROPATHY	nan	nan	0.243356	0.026667	0.612918	0.031000
KEGG_ALZHEIMERS_DISEASE	-0.467058	0.000000	0.237371	0.000000	nan	nan
KEGG_HUNTINGTONS_DISEASE	-0.467485	0.000000	0.234265	0.006329	nan	nan
HP_DILATED_CARDIOMYOPATHY	nan	nan	0.221653	0.018692	0.630726	0.002000
FORTSCHEGGER_PHF8_TARGETS_DN	-0.255267	0.037000	-0.201065	0.010560	nan	nan
BLALOCK_ALZHEIMERS_DISEASE_DN	-0.340125	0.000000	-0.227952	0.000000	nan	nan
JISON_SICKLE_CELL_DISEASE_UP	-0.363197	0.001037	-0.246233	0.014493	nan	nan
REACTOME_INFECTIOUS_DISEASE	-0.423472	0.000000	-0.256379	0.000000	nan	nan
GSE18791_CTRL_VS_NEWCASTLE_VIRUS_DC_4H_UP	-0.344760	0.003138	-0.263596	0.009547	nan	nan
OSMAN_BLADDER_CANCER_DN	-0.336170	0.000000	-0.267146	0.000000	nan	nan
WATANABE_RECTAL_CANCER_RADIOTHERAPY_RESPONSIVE_UP	-0.363195	0.024859	-0.274567	0.040951	nan	nan
SHAFFER_IRF4_TARGETS_IN_ACTIVATED_B_LYMPHOCYTE	-0.393554	0.008989	-0.293403	0.035904	nan	nan
GSE18791_CTRL_VS_NEWCASTLE_VIRUS_DC_8H_UP	-0.330549	0.008222	-0.299852	0.001242	nan	nan
GAZDA_DIAMOND_BLACKFAN_ANEMIA_ERYTHROID_DN	-0.310344	0.001004	-0.300064	0.000000	nan	nan
KYNG_RESPONSE_TO_H2O2	-0.400841	0.015012	-0.322529	0.019280	nan	nan
GSE18791_CTRL_VS_NEWCASTLE_VIRUS_DC_10H_UP	-0.303370	0.028481	-0.327363	0.000000	nan	nan
WATANABE_RECTAL_CANCER_RADIOTHERAPY_RESPONSIVE_DN	-0.381953	0.014689	-0.328710	0.010458	nan	nan
GSE18791_CTRL_VS_NEWCASTLE_VIRUS_DC_16H_UP	-0.329788	0.007277	-0.333223	0.000000	nan	nan
LI_LUNG_CANCER	-0.481780	0.003614	-0.347962	0.046448	-0.386103	0.000000
WP_ALZHEIMERS_DISEASE	-0.382814	0.039581	-0.363187	0.001383	nan	nan
BLALOCK_ALZHEIMERS_DISEASE_INCIPIENT_DN	-0.356367	0.006257	-0.366633	0.000000	nan	nan
ROSTY_CERVICAL_CANCER_PROLIFERATION_CLUSTER	-0.341836	0.037716	-0.368016	0.000000	nan	nan
JISON_SICKLE_CELL_DISEASE_DN	-0.416023	0.000000	-0.382708	0.000000	nan	nan
KYNG_RESPONSE_TO_H2O2_VIA_ERCC6_DN	-0.424105	0.040189	-0.384797	0.016552	nan	nan
SU_TESTIS	nan	nan	-0.390156	0.002692	-0.271707	0.000000
WP_PARKINSONS_DISEASE_PATHWAY	-0.429931	0.040842	-0.392037	0.016506	nan	nan
WONG_PROTEASOME_GENE_MODULE	-0.447439	0.005834	-0.397411	0.000000	-0.425707	0.000000
ZHONG_RESPONSE_TO_AZACITIDINE_AND_TSA_DN	-0.423102	0.011429	-0.401796	0.001353	-0.431392	0.000000
KEGG_AMYOTROPHIC_LATERAL_SCLEROSIS_ALS	-0.457664	0.008444	-0.424821	0.001412	nan	nan
ANDERSEN_LIVER_CANCER_KRT19_UP	-0.573476	0.000000	-0.425755	0.004155	nan	nan
VILLANUEVA_LIVER_CANCER_KRT19_UP	0.251812	0.012987	-0.457212	0.000000	nan	nan
BILD_SRC_ONCOGENIC_SIGNATURE	nan	nan	-0.468653	0.002833	-0.410392	0.033333
KEGG_CHRONIC_MYELOID_LEUKEMIA	nan	nan	-0.469593	0.000000	-0.399986	0.000000
KYNG_RESPONSE_TO_H2O2_VIA_ERCC6	-0.576794	0.010695	-0.487821	0.026746	-0.507155	0.000000
BIOCARTA_NFAT_PATHWAY	-0.457558	0.008333	-0.505455	0.000000	nan	nan
ZHAN_MULTIPLE_MYELOMA_SUBGROUPS	-0.473793	0.042735	-0.505738	0.000000	-0.553102	0.000000
BIOCARTA_GLEEVEC_PATHWAY	nan	nan	-0.602708	0.000000	-0.586174	0.000000
REACTOME_SIGNALING_BY_KIT_IN_DISEASE	nan	nan	-0.722807	0.000000	-0.745475	0.000000
REACTOME_SIGNALING_BY_FGFR2_IN_DISEASE	-0.575753	0.000000	nan	nan	-0.304140	0.000000
REACTOME_SIGNALING_BY_FGFR_IN_DISEASE	-0.497810	0.000000	nan	nan	-0.260304	0.000000

Fig. S8. Pre-ranked GSEA enrichment statistics in disease gene sets (n=375). The values were central-scaled prior to the GSEA analysis. The enrichment score (ES) is the maximum deviation from zero showing the degree to which the gene set is over-represented at the top (positive ES score) or bottom (negative ES score) of the entire ranked list of genes. Disease gene sets with the nominal p-value<0.05 and ES<-0.2 (negative enrichment) and ES>0.2 (positive enrichment) were selected and kept only those that were significant in at least two hydrophobic measures. KEGG neurodegenerative pathways are highlighted with the red squares. 'Nan' value indicates that an ES score was either between -0.2;0.2 or insignificant (p-value>0.05).

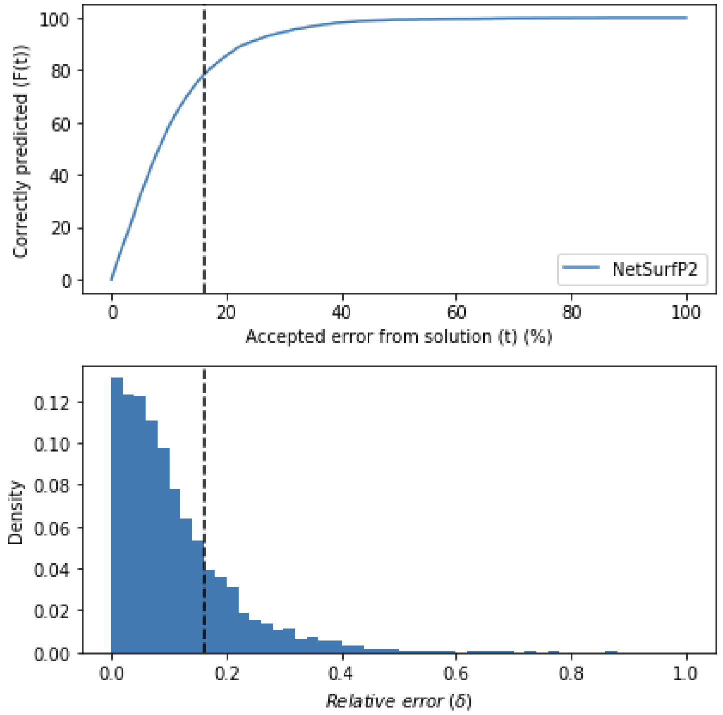

Fig. S9. An example of the relative threshold-based evaluation metric: In this case, a threshold range from 0 to 100 percent absolute error is used to derive at the curve in the top panel (Equations 1, 2, 3). For each threshold (exemplified by the black dashed horizontal line in the upper panel), the percentage of correctly predicted proteins within this threshold is calculated (Equation 4). This fraction is the density to the left of the threshold in the lower panel.

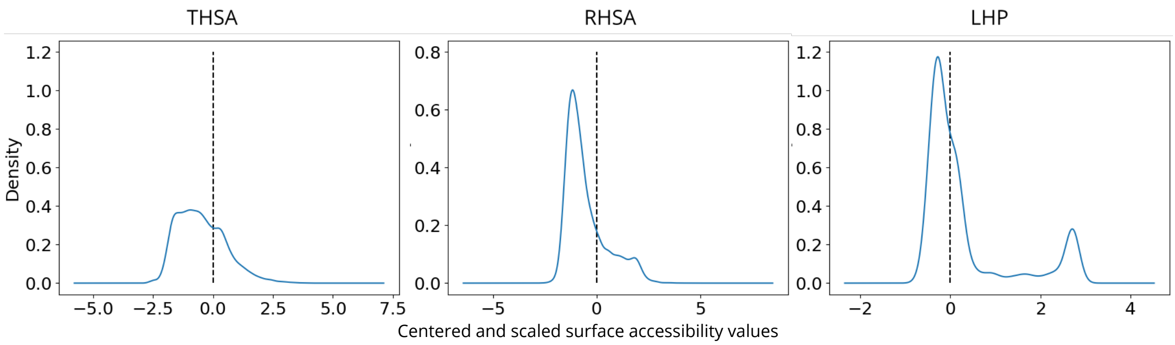

Fig. S10. Distributions of the centered and scaled values of the THSA, RHSA and LHP used for GSEA analysis. The dotted lines indicate the zero positions. Values for centering were chosen such that 0 falls in between two parts of a bimodal distribution or between the main bulk and the tail of the distribution.
